# Effect of three different attachment designs in the extrusive forces generated by thermoplastic aligners in the maxillary central incisor

**DOI:** 10.1590/2177-6709.25.3.046-053.oar

**Published:** 2020

**Authors:** Rafael Costa, Fernanda Calabró Calheiros, Rafael Yagüe Ballester, Flávia Gonçalves

**Affiliations:** 1 Universidade Ibirapuera, Faculdade de Odontologia (São Paulo/SP, Brazil).; 2 Universidade de São Paulo, Faculdade de Odontologia (São Paulo/SP, Brazil).

**Keywords:** Orthodontic extrusion, Orthodontic appliances, Biomedical technology

## Abstract

**Introduction:**

Orthodontic aligners use have increased in dentistry. The resolution of complex movements such as extrusion demands the use of attachments to reach the aimed force, but just a few studies have been developed to evaluate the biomechanical performance of the aligners and their accessories.

**Objective:**

The objective of this study was to evaluate on the three axes (X, Y and Z) the forces generated by three different attachment designs for the extrusion of the maxillary central incisor using esthetic orthodontic aligners.

**Methods:**

Three prototypes of maxillary models were developed, each one with a specific attachment inserted in the central incisor. Three aligners were manufactured for each of the three attachment designs, with 0.33-mm activation in the direction of the extrusion. An analytical device was used to evaluate the forces applied to the three axes by each aligner/attachment. The data were assessed by one-way ANOVA and Tukey’s test (α = 0.05).

**Results:**

All of the studied attachment designs could satisfactorily perform the extrusion movement. However, force intensities were different in the three designs (design 1 = 2.5 N; design 2 = 2.2 N, and design 3 = 1.1 N). Furthermore, two of the three attachment designs (designs 1 and 2) eventually exerted significant forces on the X (mesiodistal) and Y (buccopalatal) axes.

**Conclusion:**

The attachment design 3 presents the best distribution of forces for extrusion movement, generating almost null forces on X and Y axes, and lower intensity of force on the Z axis.

## RESUMO

**Introdução:**

O uso de alinhadores ortodônticos tem aumentado na Odontologia. Porém, para a realização de movimentos complexos, como a extrusão, é necessário usar *attachments* para se obter as forças desejadas. Apesar disso, há poucos estudos que tenham avaliado o desempenho biomecânico dos alinhadores e de seus *attachments*.

**Objetivo:**

O objetivo do presente estudo foi avaliar nos três eixos (X, Y e Z) as forças geradas por três diferentes *designs* de *attachments* para extrusão de um incisivo central superior usando alinhadores ortodônticos.

**Métodos:**

Foram confeccionados três modelos superiores prototipados, cada um com um tipo de *attachment*inserido no incisivo central. Três alinhadores foram fabricados para cada um dos três *designs* de *attachment*, com ativação de 0,33 mm na direção da extrusão. Foi, então, utilizado um equipamento de análise para mensurar, nos três eixos, as forças aplicadas por cada alinhador/*attachment*. Os dados foram submetidos à ANOVA de fator único e ao teste de Tukey (α?#8197;= 0,05).

**Resultados:**

Todos os *designs* de *attachments* utilizados foram capazes de exercer satisfatoriamente o movimento de extrusão. Entretanto, a intensidade das forças foi diferente nos três *designs* (*attachment 1* = 2,5 N; *attachment 2* = 2,2 N e *attachment 3*= 1,1 N). Além disso, dois dos três *designs* (*attachments 1* e *2*) exerceram forças significativas nos eixos X (mesiodistal) e Y (vestibulopalatino).

**Conclusões:**

o *design* do *attachment 3* apresentou a melhor distribuição de forças para o movimento de extrusão, sendo o mais próximo de forças nulas nos eixos X e Y e força de baixa intensidade no eixo Z.

## INTRODUCTION

In recent years, the search for esthetic solutions by orthodontic patients has increased. Aiming to achieve a less invasive, more esthetic, hygienic, and comfortable orthodontic treatment, transparent and removable orthodontic aligners have increasingly become a popular alternative to conventional treatment with metallic brackets.^1^ ^-^ ^3^

Aligner therapy is based on the sequential use of aligners to gradually move the teeth to the desired position. The forces and moments required for malocclusion correction are generated by the difference between the shape of aligners and the teeth.^4^ ^,^ ^5^ Scientific reports have demonstrated that extrusion movements with aligners are the most difficult ones;^6^ ^,^ ^7^ therefore, the use of movement accessories such as attachments is indicated.^8^

Attachments are small structures with well-defined geometry used to generate forces or moments, increasing the capacity of orthodontic aligners to move the tooth^9^ ^,^ ^10^. Although attachments have great potential, their use in dental practice is restricted because just a few studies have evaluated its mechanical behaviour,^10^ ^-^ ^12^ and there is a significant gap of information about the biomechanical performance of these accessories based on their size, geometry, and forces.^9^ ^,^ ^10^ Although the study of Dasy et al.^11^ observed higher retention force for beveled attachments than rectangular or ellipsoid ones, and Cai et al.^10^ optimized an attachment for tooth translation, no study evaluated the efficacy of attachments for tooth extrusion. 

Therefore, the present study aims to evaluate *in vitro* the forces generated on the X, Y, and Z axes, developed by different attachment designs, associated with orthodontic aligners during the extrusion movement of an maxillary central incisor. The study hypothesis is that changes in attachment design produce different loads on the tooth.

## MATERIAL AND METHODS

### Model preparation and dental software program

The maxillary model used as three-dimensional reference was obtained free of charge at Cadnav.com. The original model had 7,048 vertices, 14,028 triangles, and a file size of 908 kilobytes. The low-resolution mesh of this model would hinder its recognition by computer-aided design (CAD) software program, so the mesh was manipulated using Meshmixer (Autodesk, California, USA), and a more refined design was then obtained and recognized by the dental software (CAD/CAM OrthoAnalyzer 2013, 3Shape, Copenhagen, Denmark), which was used to simulate orthodontic movements.

The model was parameterized in the following stages: segmentation of the tooth and determination of the limit between tooth and gingiva; identification of the mesiodistal limit of each tooth; and determination of the long axis of the tooth. In the parameterized model, a 0.33-mm extrusion movement was simulated on the Z-axis of the right maxillary incisor, considering the Z axis to be equal to the long axis of the tooth. 

### Development and insertion of the attachments

**Figure 1:**
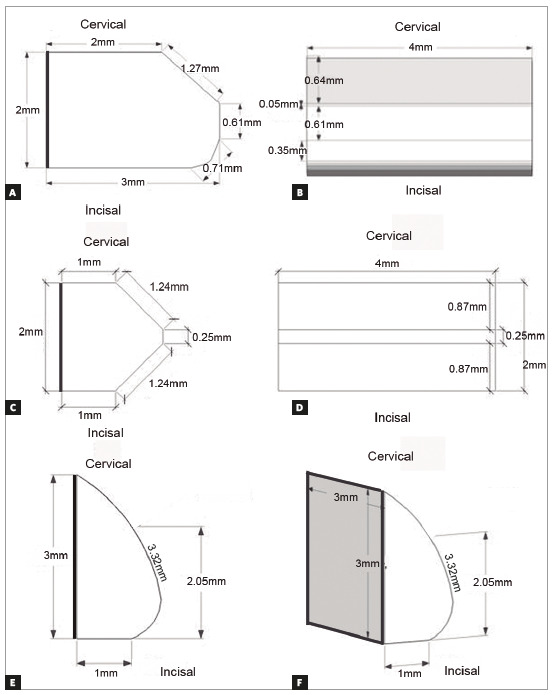
Attachment 1 in lateral view (A) and frontal view (B). Attachment 2 in lateral view (C) and frontal view (D). Attachment 3 in lateral view (E) and isometric view (F).

Three attachment designs were developed using the Sketchup software (Trimble, California, USA), as shown in Figure 1. Although the designs were slightly based on the conventional rectangular, bevelled and ellipsoid conventional attachments, they represent a new geometry and concept, aiming to optimize attachments design for free of patents use. The marginal faces of attachments are larger than conventional models, increasing their prominence, and a retentive inclined plane in the vestibular face of the attachment was created to increase its active area. 

The first attachment geometry (Figs 1A and 1B) consisted of a rectangle with 8 mm² on its gingival face and a 3-mm thickness from the dental surface to the frontal face, to provide aligner retention.

The second attachment geometry (Figs 1C and 1D) was designed for force application at 45^o^ from the movement, thus facilitating aligner insertion. It was designed from a 2*x*4*x*1 mm^3^ cuboid, associated with two 0.87*x*4 mm^2^ rectangular planes angled at 45^o^ with the cuboid surface. 

The third attachment geometry (Figs 1E and 1F) presented a frontal face without edges and less protrusive, with a vestibular length of 3.32 mm. This geometry was presumably more comfortable than the others.

All the attachments were inserted in the central area of the right maxillary incisor using the mean point between the following guidelines: incisal edge, cementoenamel junction and the proximal contact points. 

### Model prototype and aligner manufacture

**Figure 2:**
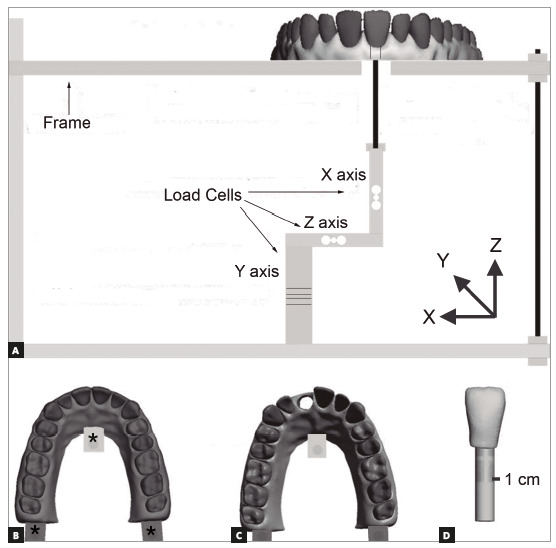
A) Force measurement device, with three load cells coupled. B) Three points for model fixation in the device. C) Cylindrical cut for connection between the teeth and the device. D) Cylindrical shaft created in the central incisor.

The models were imported into the Meshmixer software to be prototyped. Two models were prototyped for each attachment. The first model (phase 0), with the teeth in the original position, was used to calibrate the device for force measurements in the initial position. The second model (phase 1) with a 0.33-mm extrusion of tooth #11 on the Z axis, was used to develop the aligner in which the forces would be measured. In both models, a cylindrical section was performed to allow the connection of tooth #11 to the device for force measurements (Fig 2C). The prototypes were printed in a 3D printer (model ZprinterProJet CJP360, 3Dsystems, Valencia, USA), with a resolution of 2 layers/min and interlayer resolution of 0.08 mm. It was used a monochromatic high-performance print material, composed by zp131 Powder (Z Corporation, Burlington, USA).

Aligners were made for each prototyped model using BioStar (Scheu, Iserlohn, Germany) with a positive pressure of 6 bar. Polyacetal DH aligner (Dhpro, Paraná, Brazil), with thickness 0.75 mm and d = 125 mm, was used to build the aligners. After acetate cooling, the edges of the aligner were cut with an HM carbide cutter (Scheu, Iserlohn, Germany) and polished with Finishing Set (Scheu, Iserlohn, Germany). 

### Force measurements

**Figure 3:**
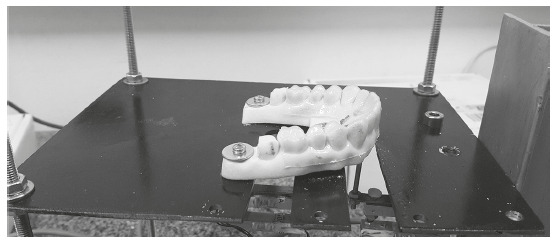
3D-printed model, with thermoplastic aligner fixed in the frame.

Figure 2A shows a schematic view of the device used to measure the forces generated by the aligners. The device was specifically developed for the present study. Three one-dimensional load cells, with a capacity between 0.01 and 7.5 N and a precision of 0.01 N (Diamond, Hong Kong, China), calculated the load on the X, Y, and Z axes. The device was calibrated by weighing the aligners in each axis to verify whether only pure forces were measured, thus ensuring that torque would not affect the outcomes. A frame allowed the fixation and adjustment of the model, as well as the connection of the right central incisor to the measurement device, as shown in Figure 2. The model was screwed to the frame in three points, as highlighted in Figure 2B, and no movement of the model was observed during the test. Figure 3 shows the model fixed to the frame. 

The maxillary central incisor, kept in position in the model, was connected to the measurement device through a metal rod abutment (Fig 2D). The metal rod was fixed in the maxillary central incisor using a ruler to maintain the vertical axis previously established, and it was fixed to the device with a screw nut. The difference between the cylindrical van in the model and the cylindrical rod of the upper central incisor was around 1 mm. With phase 0 aligner in position, the model position was adjusted until the load cells showed a load of zero, when the auto-zero function was activated. 

Three aligners were fabricated for each attachment model. Each aligner was measured three times (27 measurements). The values obtained by the force measurement device are expressed in grams-force (gf) and were converted to Newton, multiplying it for 9.8. The resultant force and the angle of inclination () of the resultant force on the Z axis were calculated according to the forces components observed in the three axes. 

The data were checked for normality (Shapiro-Wilk test) and homoscedasticity (Bartlett’s test), and assessed by one-way ANOVA and Tukey’s test, with a 95% significance level (α=0.05). 

## RESULTS

Data on the forces generated on the X, Y, and Z axes and the resultant force are shown in Table 1. The X-axis represents the forces towards the mesial (positive) and distal (negative) direction of the tooth. Note that the assessed attachments showed significant differences between them: attachment 2 produced the lowest force and had the opposite direction, when compared to the other attachments. 

**Table 1:** Mean and standard deviation of the forces (N) developed in the three axes (X, Y and Z), the resultant force from the 3 axes and from X and Y axes, and the angle of the resultant inclination, using orthodontic aligners associated to three attachments designs.

	Force (N)				Angle of the resultant inclination in Z axis (^o^)	
	X axis	Y axis	Z axis	Resultant force from the 3 axes	Resultant force from X and Y axes	
Attachment 1	0.47 ± 0.06^a^	0.25 ± 0.02^b^	2.53 ± 0.02^a^	2.59 ± 0.01^a^	0.53 ± 0.04^b^	11.9 ± 1.1^c^
Attachment 2	-0.17 ± 0.02^c^	-1.27± 0.05^a^	2.17 ± 0.01^b^	2.52 ± 0.03^b^	1.28 ± 0.05^a^	30.6 ± 1.1^a^
Attachment 3	0.35 ± 0.01^b^	0.26 ± 0.03^b^	1.12 ± 0.01^c^	1.20 ± 0.01^c^	0.44 ± 0.01^b^	21.3 ± 0.2^b^

Similar superscript letters in one column indicate the absence of statistical differences (α = 0.05).

The Y-axis represents the force towards the buccopalatal direction, and it was positive for the buccal direction and negative for the palatal one. Forces in attachments 1 and 3 were towards the buccal direction and showed no statistically significant difference between them, but they both generated forces of significant lower intensity and inverse direction than did attachment 2, whose forces were towards the palatal direction. 

The Z-axis represents the extrusion (positive) and intrusion (negative) movements of the tooth. As expected, the three attachments showed positive forces, with statistically significant differences in their intensity. 

**Figure 4:**
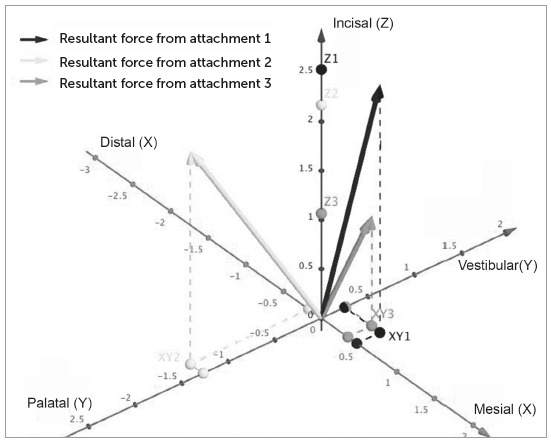
Resultant forces analyzed on the, X, Y, and Z axes.

The three attachments revealed significant differences in the resultant force, as illustrated in Figure 4. Note that attachment 1 had the highest resultant force. The angles of inclination of the resultant force on the Z-axis are shown in Table 1. Attachment 1 had the lowest angular deviation from the Z-axis, and attachment 2 showed the highest angular deviation - these angular deviations were statistically different. However, when the resultants forces from the X and Y axes are observed, attachment 3 showed the significant lowest deviation from Z, whereas attachment 2 had the highest deviation. 

## DISCUSSION

Different attachment geometries provide different loadings on the maxillary central incisor, regarding the resultant force and forces on the Z and X axes. The forces generated on the Y axis were similar between attachment models 1 and 3. Among the three attachments, attachment 2 differed more sharply from the other two, not only in its force intensity on all axes, but also in its force direction on the X and Y axes. In fact, the plane designed to incline the force to 45^o^ had an excessive effect. 

On the X axis, attachment model 2 generated 0.17 N in the negative direction, which is not considered high enough to perform an orthodontic movement - the literature recommends that at least 0.35 N should be applied to perform this type of movement.^13^ Conversely, attachments 1 and 3 showed forces in the distal direction with different intensities: attachment 1 exceeded the 0.35 N proposed in the literature, while attachment 3 generated exactly 0.35 N, being on the threshold of tooth movement. The presence of these mesiodistal forces can be attributed to jittering that could develop between the aligner and the attachment; differences in the number of teeth on which the aligner is supported on each side of the moved teeth; and morphological differences between the mesial and distal surfaces of the moved teeth. 

Regarding the Y-axis, attachment 2 generated a negative force of 1.27 N, favouring palatal tooth movement. Even with the opposite force developed by the aligner, the high force magnitude on the Y-axis exceeded 0.35 N; which, according to the literature, would be enough for orthodontic movement. However, attachments 1 and 3 showed similar forces on the Y axis: 0.25 and 0.26 N, respectively, both in a positive direction and not able to promote tooth movement. It is believed that the force in attachment 2 was towards the palatal direction due to its pyramidal geometry. In this model, a force exerted on the Z-axis when applied to the plane at 45^o^ from the movement direction can be decomposed into a force on the Y-axis, which would help explain the palatal direction of the forces generated in this attachment model. Another explanation is the plane inclined towards the incisal surface, which differs significantly from the other attachment designs and could develop intrusive forces, decreasing forces on the Z axis and increasing them on the Y-axis.

To develop an design for pure extrusion movement, the attachment should ideally generate null forces on the X and Y axes. None of the analyzed attachments was able to exercise null forces on any of the axes X and Y. The performance of attachment 3 was close to that, since its forces were not enough for tooth movement on the Y axis and similar to threshold forces on the X-axis, with the lowest resultant force on the X and Y axes, being the most promising design. Although showing higher angular deviation from the Z-axis than did attachment 1, this was mainly due to the lower intensity of the force on the Z axis, rather than the intensity of the other components of the resultant force. Although these attachments designs are unique and represent a new geometry and concept, aiming to optimize attachments design for use free of patents, to date, no studies have been conducted on attachment designs for extrusion movement with aligners in the maxillary central incisor, nor with the conventional attachment. Just one study has evaluated the attachment geometry for canine translation.^12^ Hence, it is not possible to compare those attachment designs with the ones developed in this study, since the dental movements evaluated are different and it is necessary to optimize the attachment design for each dental movement.

Regarding the forces on the Z axis, attachment 1 had the highest force intensity, due to its rectangular face at an angle of 90^o^ in the force direction and possibly due to its larger protuberance than the other attachments, allowing for a bigger contact area between the aligner and the attachment. Attachment 2, even at an angle of 45^o^ with the Z axis, revealed an intermediary force, possibly because of its 1*x*4 mm² rectangular face in the cervical area of the teeth (Fig 1C). For having a rounded geometry, with no edges, attachment 3 showed a smaller contact area with the Z axis, and generated a lower force on it, in addition to a lower inclination of the attachment on its cervical face, showing a more vertical shape, allowing force intensity to decrease on the Z axis. The analysis of retentive force of conventional aligners has pointed that the ellipsoid attachment presented lower retention than rectangular or bevelled designs,^11^ which besides of geometric and size differences can be in accordance to the present study, where the rounded attachment (design 3) presented lower forces in Z axis.^11^ There was no contact between the incisal edge and the aligner to perform an extrusion movement; so there was not a normal force (opposite to the movement) which opposed the extrusion movement. It is known in the literature that a minimum load of 0.35 N and maximum load of 0.6 N are needed for tooth extrusion, but the intensity varies according to root shape and size.^13^ In this study, all attachments generated a force 3 to 7.5 times higher than the minimum threshold for extrusion movement, and 2 to 4 times higher than the maximum force. A limitation of this study concerns the fact that, in the tested model, all teeth, except for the right incisor, were fixed to each other and to the model. So, they exercised a rigid anchorage to move the right incisor, which could increase the forces on this tooth. However, in clinical situations, all teeth used to anchor the movement are free and the periodontal ligament can release part of the generated stress, and lower force intensity can be developed to extrude the tooth.

Furthermore, in the present study, the forces were measured immediately after aligner insertion, but it has been reported that significant stress relaxation is observed in the aligners in the first eight hours after instalation,^14^ what would decrease the forces transferred to the tooth. However, more studies are needed to observe the behaviour of these aligners associated with the attachments over a long term. These higher forces should be viewed with caution, as excessive forces applied to orthodontic movement can cause injuries to the supportive tissues. Several studies have shown that orthodontic aligners promote root resorption that is similar to or lower than that generated by conventional brackets.^15,16^ Hemanth et al.^17^ ^,^ ^18^, using finite element method (FEM), observed that dental extrusion movement causes tensile stress in the dental apical area and compressive stress in the cervical portion of the root, which is in accordance to the system proposed by Proffit et al.^13^ These effects may be maximized with the increase of forces generated during an orthodontic treatment; however, further studies are needed to evaluate these biological effects.

By analyzing the resultant force, one can observe that among the three assessed designs, attachment 3 is the one with the lowest force intensity on the X and Y axes, although the intensity is twice as high as the maximum load recommended in the literature, and its biological effects remain unknown. In addition to the mechanical aspects described, some other advantages include the rounded edges, as they help lower retention, facilitating aligner removal. Attachment 2 generated excessive force on the Y-axis; consequently, it cannot be indicated for pure extrusion movement, but its application could be investigated when the tooth also requires an inclination of the palatal plane. Finally, attachment 1 is similar to attachment 3 on the Y axis, with the lowest angular deviation from the resultant force, and exercises higher forces on the Z and X axes, which would be undesirable in clinical application.

Therefore, the results obtained in this study do not allow the direct indication of these attachments for immediate clinical use, but the proposed method is simple and promising for the biomechanical evaluation of attachments in orthodontic movements with aligners. A load cell, with a capacity between 0.01 and 7.5 N and a precision of 0.01 N, was used to build the measuring force device, its precision is higher than other orthodontic force tester describe in the literature,^19^ and enough for evaluating orthodontic forces, being a simple tool to optimize attachments design. These results may contribute to scientific advances and knowledge of biomechanical attachments, leading to the optimization of new attachments and improving treatment predictability regarding extrusion movements. 

## CONCLUSION

Despite the limitation of this study, it may be concluded that: (1) different attachment geometries generate forces with significantly different intensity and direction; (2) attachment 3 had the best mechanical performance among the three models evaluated. Its force on the Z axis was enough for orthodontic extrusion and significantly lower than that of the other attachments, although the force intensity can still be considered high; forces on the Y axis are not enough for moving teeth and in X axis is on the threshold of tooth movement; (3) further studies are needed to improve attachment 3 design and to evaluate its biological effect on the supportive tissue. 

## References

[1] Weir T (2017). Clear aligners in orthodontic treatment. Aust Dent J.

[2] White DW, Julien KC, Jacob H, Campbell PM, Buschang PH (2017). Discomfort associated with Invisalign and traditional brackets A randomized, prospective trial. Angle Orthod.

[3] Zheng M, Liu R, Ni Z, Yu Z (2017). Efficiency, effectiveness and treatment stability of clear aligners a systematic review and meta-analysis. Orthod Craniofac Res.

[4] Beers AC, Choi W, Pavlovskaia E (2003). Computer-assisted treatment planning and analysis. Orthod Craniofac Res.

[5] Wong BH (2002). Invisalign A to Z. Am J Orthod Dentofacial Orthop.

[6] Kravitz ND, Kusnoto B, BeGole E, Obrez A, Agran B (2009). How well does Invisalign work A prospective clinical study evaluating the efficacy of tooth movement with Invisalign. Am J Orthod Dentofacial Orthop.

[7] Rossini G, Parrini S, Castroflorio T, Deregibus A, Debernardi CL (2015). Efficacy of clear aligners in controlling orthodontic tooth movement a systematic review. Angle Orthod.

[8] Boyd RL (2008). Esthetic orthodontic treatment using the Invisalign appliance for moderate to complex malocclusions. J Dent Educ.

[9] Barone S, Paoli A, Razionale AV, Savignano R (2017). Computational design and engineering of polymeric orthodontic aligners. Int J Numer Method Biomed Eng.

[10] Cai Y, He B, Yang X, Yao J (2015). Optimization of configuration of attachment in tooth translation with transparent tooth correction by appropriate moment-to-force ratios Biomechanical analysis. Biomed Mater Eng.

[11] Dasy H, Dasy A, Asatrian G, Rozsa N, Lee HF, Kwak JH (2015). Effects of variable attachment shapes and aligner material on aligner retention. Angle Orthod.

[12] Gomez JP, Pena FM, Martinez V, Giraldo DC, Cardona CI (2015). Initial force systems during bodily tooth movement with plastic aligners and composite attachments A three-dimensional finite element analysis. Angle Orthod.

[13] Proffit WR, Fields HW, Sarver DM (2014). Contemporary orthodontics.

[14] Lombardo L, Martines E, Mazzanti V, Arreghini A, Mollica F, Siciliani G (2017). Stress relaxation properties of four orthodontic aligner materials a 24-hour in vitro study. Angle Orthod.

[15] Elhaddaoui R, Qoraich HS, Bahije L, Zaoui F (2017). Orthodontic aligners and root resorption a systematic review. Int Orthod.

[16] Gay G, Ravera S, Castroflorio T, Garino F, Rossini G, Parrini S (2017). Root resorption during orthodontic treatment with Invisalign(r) a radiometric study. Prog Orthod.

[17] Hemanth M, Deoli S, Raghuveer HP, Rani MS, Hegde C, Vedavathi B (2015). Stress induced in the periodontal ligament under orthodontic loading (Part I) A Finite element method study using linear analysis. J Int Oral Health.

[18] Hemanth M, Raghuveer HP, Rani MS, Hegde C, Kabbur KJ, Chaithra D (2015). An Analysis of the stress induced in the periodontal ligament during extrusion and rotation movements- Part II a comparison of linear vs nonlinear FEM Study. J Contemp Dent Pract.

[19] Chen J, Isikbay SC, Brizendine EJ (2010). Quantification of three-dimensional orthodontic force systems of T-loop archwires. Angle Orthod.

